# Dynamic aphasia as an early sign of corticobasal degeneration: Clinico-radio-pathological correlation

**DOI:** 10.1016/j.ensci.2024.100526

**Published:** 2024-09-04

**Authors:** Masanori Kurihara, Akira Arakawa, Aya Midori Tokumaru, Tomoyasu Matsubara, Hiroto Eguchi, Yasushi Shimo, Masato Hasegawa, Kazutomi Kanemaru, Katsuhiko Takeda, Atsushi Iwata, Shigeo Murayama, Yuko Saito

**Affiliations:** a Department of Neurology, Tokyo Metropolitan Institute for Geriatrics and Gerontology, Tokyo, Japan; b Department of Neuropathology (Brain Bank for Aging Research), Tokyo Metropolitan Institute for Geriatrics and Gerontology, Tokyo, Japan; c Department of Diagnostic Radiology, Tokyo Metropolitan Institute for Geriatrics and Gerontology, Tokyo, Japan; d Department of Neurology, Juntendo University Nerima Hospital, Tokyo, Japan; e Department of Brain and Neurosciences, Tokyo Metropolitan Institute of Medical Science, Tokyo, Japan; f Bunkyo Cognitive Neuroscience Laboratory, Tokyo, Japan; g Brain Bank for Neurodevelopmental, Neurological and Psychiatric Disorders, Molecular Research Center for Children's Mental Development, United Graduate School of Child Development, Osaka University, Osaka, Japan

**Keywords:** Corticobasal degeneration, Frontal Aslant tract, Supplementary motor area, Dynamic aphasia, Verbal adynamia, White matter

## Abstract

A 72-year-old man presented with a 6-month history of decreased voluntary speech. Sparse speech and decreased word fluency were observed. Articulation, naming, comprehension, and repetition were preserved. Agrammatism and paraphasia were not observed. These characteristics matched those reported as dynamic aphasia. Other findings were mild behavioral symptoms, recent memory impairment, and right hemiparkinsonism. The patient‘s voluntary speech continued to reduce and behavioral symptoms progressed. Brain MRI including voxel-based morphometric analysis showed left-dominant white matter volume reduction in the frontal lobe including those between the left supplementary motor area (SMA)/preSMA and the frontal operculum, likely involving the frontal aslant tract (FAT). The patient became completely mute after two years from disease onset and died of aspiration pneumonia. The neuropathological diagnosis was corticobasal degeneration (CBD). This case suggests that dynamic aphasia may be the initial sign of CBD and that early involvement of left FAT may be responsible for this feature.

## Highlights

• A patient with corticobasal degeneration (CBD) presented with dynamic aphasia.
• MRI indicated early degeneration of the left frontal aslant tract (FAT).
• Symptoms progressed to CBD-frontal behavioral-spatial syndrome.
• CBD was confirmed pathologically involving both gray and white matters.
• CBD can present with dynamic aphasia and degeneration of FAT may be involved.

Corticobasal degeneration (CBD) is a neurodegenerative disease characterized by cortical and striatal 4-repeat-tau-positive inclusions in the neurons and glial cells, including astrocytic plaques [1]. Clinical presentations of CBD include corticobasal syndrome (CBS), frontal behavioral-spatial syndrome (FBS), and non-fluent/agrammatic variants of primary progressive aphasia (naPPA) [2]. naPPA is characterized by effortful non-fluent speech due to grammatical impairment and speech apraxia and is associated with cortical atrophy in the left frontal operculum [3]. Dynamic aphasia (verbal adynamia) is characterized by disproportionate impairment of propositional speech (narrative/spontaneous verbal expression) and verbal fluency despite relatively preserved ability to produce speech in naming and repetition [4,5]. However, it was unknown if dynamic aphasia is observed in pathologically confirmed CBD. Moreover, although disease pathology involves white matter abnormalities that can be detected using brain magnetic resonance imaging (MRI) [6,7], the association between these white matter abnormalities and the associated symptoms remains unclear.

The frontal aslant tract (FAT) is a short connection within the frontal white matter connecting the supplementary motor area (SMA)/preSMA and the pars opercularis/pars triangularis of the inferior frontal gyrus (Broca’s area in the left hemisphere) [[8], [9], [10]]. The left FAT is vital for verbal initiation and fluency which could be impaired by electrical stimulation or chronic degeneration [[8], [9], [10]]. However, the association between the degeneration of the FAT and isolated impaired verbal initiation and fluency in patients with CBD remains unknown.

Herein, we describe a patient with pathologically confirmed CBD initially presenting with impaired verbal initiation and fluency compatible with dynamic aphasia, whose MRI results suggested early degeneration of the left FAT.

A 72-year-old right-handed man presented with a six-month history of gradually declining spontaneous speech and apathy. During this period, he stopped working but continued to walk around his neighborhood and engaged in other daily activities. Sparse speech and decreased word fluency were evident upon initial assessment. Nevertheless, articulation, naming, comprehension, and repetition abilities remained. Agrammatism or paraphasia was not observed, while mild recent memory impairment and right-sided hemiparkinsonism were noted. Brain MRI revealed enlargement of the left lateral ventricle and mild widening of the left frontal sulcus. After initiating treatment with levodopa/carbidopa, behavioral symptoms emerged and his parkinsonism did not improve. Subsequent discontinuation of levodopa/carvidopa improved the behavioral symptoms, but they still persisted. Based on these findings, CBD-FBS was suspected.

**Fig. 1:**
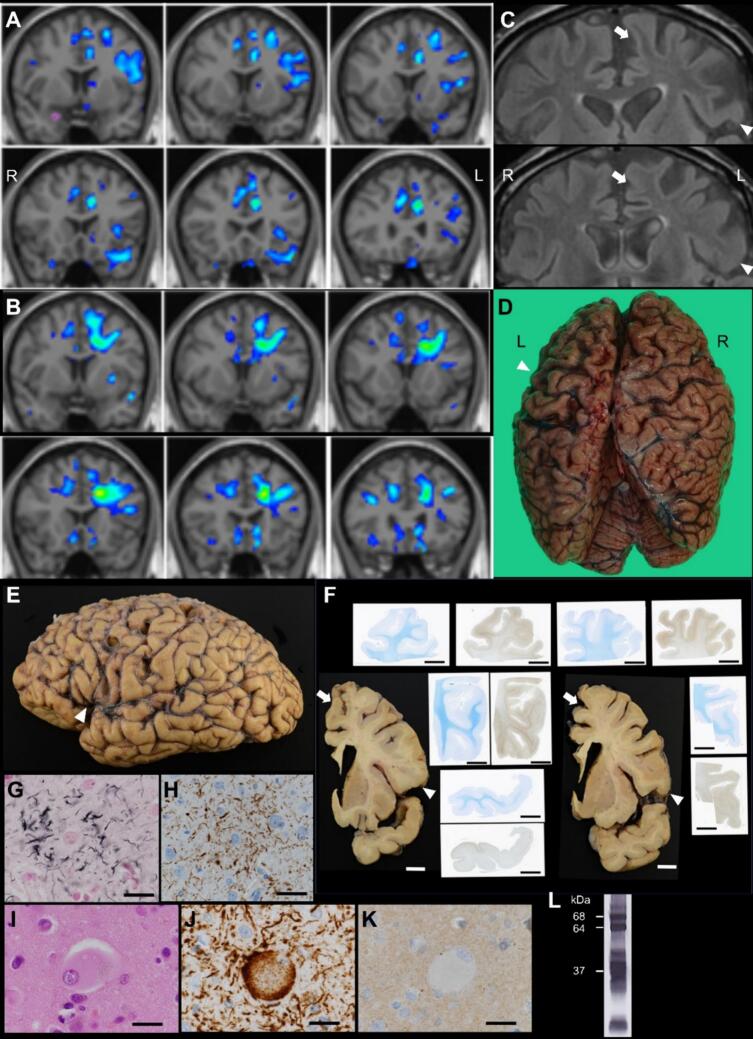
Radiological and neuropathological results. (A, B) Gray (A) and white (B) matter volume reduction analysis based on 3D T1-weighted magnetic resonance imaging (MRI) (one year from disease onset). (A) Gray matter volume reduction was suspected in the left anterior cingulate gyrus, left frontal operculum, and part of the left superior frontal gyrus. (B) White matter volume reduction was observed in the left frontal lobe in the subcortical white matter of the superior and middle frontal gyrus (including the supplementary motor area [SMA]/preSMA) and deep white matter between the SMA/preSMA and the frontal operculum. (C) Fluid-attenuated inversion recovery images showed mild high-intensity signals in the left frontal white matter (arrow: medial superior frontal gyrus, arrowhead: posterior inferior frontal gyrus). (D) Macroscopic image of the autopsied brain before fixation (3 years after disease onset). At autopsy, strong left-dominant frontal lobe atrophy and left parietal lobe atrophy were observed at the convexity. (E) Macroscopic image of the formalin-fixed left-brain hemisphere. Convexity atrophy was observed in the frontal and parietal lobes. Brain atrophy was also observed at the lower precentral gyrus, the frontal operculum (posterior inferior frontal gyrus; arrowhead), and the anterior superior temporal gyrus. (F) Macroscopic and semimacroscopic images of sagittal sections of the left hemisphere. Frontal volume reduction was observed in the gray and white matter, including the white matter between the medial superior frontal gyrus (arrow) and the frontal operculum (arrowhead). Discoloration of the white matter was also observed. Klüver–Barrera staining showed an obscured corticomedullary junction. Phosphorylated tau (AT8) immunostaining showed numerous immunoreactive structures, mostly accentuated in the corticomedullary junction. The distribution of these tau inclusions was mostly accentuated in these gyri, compared to other cortical areas including the temporal tip. (G-K) Microscopic images of the pars opercularis. Astrocytic plaque was observed (G: Gallyas–Braak stain; H: AT8 immunostaining). Ballooned neuron with granulovacuolar degeneration was observed (I; hematoxylin and eosin stain). These structures were immunoreactive for 4-repeat tau (RD4) (J), but not for 3-repeat tau (RD3) (K). (L) Western blot analysis of the sarkosyl-insoluble fraction. Two bands at 68 and 64 kDa corresponding to 4-repeat-tau as well as bands around 37 kDa corresponding to the C-terminal fragment are observed, which are compatible with CBD. R: right, L: left. Scale bars: 1 cm (F), 25 μm (G-K).

Although the patient’s voluntary speech continued to decline, grammatical or articulation impairments were not noted. Follow-up MRI, including voxel-based morphometric analysis, suggested only a mild reduction in gray matter volume in the left frontal lobe (Fig. 1A). However, reduced white matter volume was observed in the left frontal lobe, including the area between the left SMA/preSMA and frontal operculum (Fig. 1B). Mild high-intensity signals were also observed on fluid-attenuated inversion recovery (FLAIR) images in the left frontal white matter (Fig. 1C). The patient became completely mute, and using utensils with his right hand became difficult two years after disease onset. Three years after the disease onset, the patient exhibited akinetic mutism and died of aspiration pneumonia. Autopsy was performed after permission from the patient’s family.

The brain weighed 1,020 g. During autopsy, pronounced left-dominant frontal lobe atrophy and left parietal lobe atrophy were observed at the convexity (Fig. 1D) and in the lower precentral gyrus, frontal operculum, and anterior superior temporal gyrus (Fig. 1E). Sagittal sections showed frontal volume reduction in both gray and white matter, including the white matter between the medial superior frontal gyrus and frontal operculum, in addition to white matter discoloration. Semimacroscopic images revealed intense phosphorylated tau staining in the gyri (Fig. 1F). Microscopic examination revealed astrocytic plaques and ballooned neurons, positive for phosphorylated tau and 4-repeat tau, present in the left medial superior frontal gyrus, frontal operculum, and subcortical white matter, which was consistent with the observed macroscopic changes (Fig. 1G-K). Western blotting analysis of sarkosyl-insoluble tau revealed bands of approximately 37 kDa (Fig. 1L). Based on these findings, the patient was pathologically diagnosed with CBD.

In this case, the characteristics of the patient’s initial speech impairment matched those reported for dynamic aphasia [4,5]. Although the patient exhibited apathy, his spontaneous activity, excluding speech, was relatively preserved. Despite the suspected loss of mild gray matter volume in the left frontal operculum, the patient did not exhibit characteristics of naPPA, such as grammatical impairment or speech apraxia [3]. His characteristics were similar to those in previous reports of impaired verbal initiation due to left SMA destruction (sometimes referred to as SMA aphasia) or dynamic aphasia reported in frontotemporal dementia, progressive supranuclear palsy, and CBS [4,5]. Other reports of patients with similar presentations have suggested that impairment of the left inferior frontal cortex [4,5] or left superior frontal cortex (including the SMA) [4,11] may be related to reduced speech based on gray matter volumetric analysis, fluorodeoxyglucose positron emission tomography, or perfusion single-photon emission computed tomography.

The brain MRI in our patient suggested white matter degeneration. Visual interpretation revealed enlargement of the left lateral ventricle and mild widening of the left frontal sulcus. White matter volume reduction analysis using SPM8 plus DARTEL [7] suggested volume loss in the left frontal white matter, including the subcortical area of the SMA/preSMA and the area between the SMA/preSMA and the frontal operculum. Furthermore, the FLAIR images showed high-intensity signals in these white matter areas. The anatomical distribution of these white matter abnormalities includes the reported distribution of FAT [9]. While CBD is a common pathological background of naPPA [3], the left FAT is associated with verbal initiation and fluency but not with other features of naPPA [8]. Based on the gray and white matter volumetric analysis in this case and in previous literature, decreased white matter volume, including the left FAT, may also be responsible for the reduced spontaneous speech, known as dynamic aphasia in CBD. However, limitation of this report is that detailed MRI to evaluate the involvement of specific white matter tracts was unavailable and future studies using such tract specific analyses are needed to prove our hypothesis.

In conclusion, dynamic aphasia may be an initial sign of CBD. Early disease involvement of the left frontal white matter, including the FAT, may be responsible for this manifestation. Further studies, including white matter analyses, are necessary to elucidate the clinic-radio-pathological correlations in CBD.

## Ethical statement

Written informed consent for autopsy and reporting was obtained from the patient’s family.

## Funding

This study was partially supported by 10.13039/501100001691JSPS KAKENHI Grant No. JP23K14789 (M. Kurihara); JP22H04923 (CoBiA) (S. Murayama, Y. Saito); JP22K15740 (T. Matsubara); MHLW Research on rare and intractable diseases Program Grant No. JPMH23FC1008 (Y.Saito); and 10.13039/100009619AMED Grant No. JP23dk0207057h0002 (A. Iwata) and JP21wm0425019 (S. Murayama, Y.Saito).

## CRediT authorship contribution statement

**Masanori Kurihara:** Writing – original draft, Visualization, Investigation, Funding acquisition, Conceptualization. **Akira Arakawa:** Writing – review & editing, Visualization, Investigation. **Aya Midori Tokumaru:** Writing – review & editing, Investigation. **Tomoyasu Matsubara:** Writing – review & editing, Investigation, Funding acquisition. **Hiroto Eguchi:** Investigation. **Yasushi Shimo:** Investigation. **Masato Hasegawa:** Investigation. **Kazutomi Kanemaru:** Investigation. **Katsuhiko Takeda:** Writing – review & editing, Conceptualization. **Atsushi Iwata:** Supervision, Investigation, Funding acquisition. **Shigeo Murayama:** Investigation, Funding acquisition. **Yuko Saito:** Writing – review & editing, Supervision, Investigation, Funding acquisition.

## Declaration of competing interest

None.
